# High-resolution terrestrial climate, bioclimate and vegetation for the last 120,000 years

**DOI:** 10.1038/s41597-020-0552-1

**Published:** 2020-07-14

**Authors:** Robert M. Beyer, Mario Krapp, Andrea Manica

**Affiliations:** Department of Zoology, University of Cambridge, Downing Street, Cambridge, CB2 3EJ United Kingdom

**Keywords:** Palaeoecology, Palaeoclimate

## Abstract

The variability of climate has profoundly impacted a wide range of macroecological processes in the Late Quaternary. Our understanding of these has greatly benefited from palaeoclimate simulations, however, high-quality reconstructions of ecologically relevant climatic variables have thus far been limited to a few selected time periods. Here, we present a 0.5° resolution bias-corrected dataset of global monthly temperature, precipitation, cloud cover, relative humidity and wind speed, 17 bioclimatic variables, annual net primary productivity, leaf area index and biomes, covering the last 120,000 years at a temporal resolution of 1,000–2,000 years. We combined medium-resolution HadCM3 climate simulations of the last 120,000 years with high-resolution HadAM3H simulations of the last 21,000 years, and modern-era instrumental data. This allows for the temporal variability of small-scale features whilst ensuring consistency with observed climate. Our data make it possible to perform continuous-time analyses at a high spatial resolution for a wide range of climatic and ecological applications - such as habitat and species distribution modelling, dispersal and extinction processes, biogeography and bioanthropology.

## Abstract (summary)

**Table:** 

Measurement(s)	temperature • precipitation process • vegetation layer • atmospheric wind speed • cloud • humidity
Technology Type(s)	computational modeling technique
Factor Type(s)	geographic location • temporal interval
Sample Characteristic - Environment	climate system
Sample Characteristic - Location	Earth (planet)

Machine-accessible metadata file describing the reported data: 10.6084/m9.figshare.12436484

## Background & Summary

Global climate in the Late Quaternary has played a major role in the formation of a wide range of macroecological patterns. Reconstructing climatic conditions has been crucial in advancing our understanding of the spatial and temporal dynamics of these processes, ranging from the distribution of species ranges^1,2^ and extinctions^3^, to early human expansions^4^ and population genetics^5^.

Climate models can provide the spatial coverage that localised empirical reconstructions are lacking, yet, currently available simulation data for the Late Pleistocene and the Holocene suffer from one of two drawbacks that limit their use for ecological applications. On the one hand, a number of equilibrium and transient simulations, from general circulation models (e.g. HadCM3^6^) or earth system models of intermediate complexity (e.g. LOVECLIM^4^), provide reconstructions at a high temporal resolution, however, the relatively low spatial resolution of the simulated data, and significant biases when compared to empirical observations^7^, make additional curating of model outputs necessary in order to generate ecologically meaningful data. On the other hand, several high-resolution and bias-corrected palaeoclimate datasets provide climatic variables in great spatial detail, but their temporal coverage of the Late Pleistocene and the Holocene is usually limited to a few snapshots of key time periods. A number of these datasets have been made available in readily accessible formats since the mid-2000s, and have since been used extensively in ecological applications: the ecoClimate database^8^ provides data for the Mid-Holocene (~6,000 BP) and the Last Glacial Maximum (~21,000 BP); WorldClim^9^ contains an additional reconstruction of the Last Interglacial Period (~130,000 BP); paleoClim^10^ covers the last 21,000 years.

Here, we fill the gap between these two types of available data, by deriving a high-resolution (0.5°) bias-corrected time series of global terrestrial climate and vegetation data covering the last 120,000 years. Gridded reconstructions (Table 1) are available at 2,000 year time steps between 120,000 and 22,000 BP, and 1,000 year time steps between 21,000 BP and the pre-industrial modern era. Our data include monthly temperature and precipitation, and 17 bioclimatic variables, which have been used extensively in species distribution models (e.g.^11^). We also provide monthly cloudiness, relative humidity and wind speed (which can be used to derive various measures of apparent temperature), as well as reconstructions of global biomes, leaf area index and net primary productivity. Our data show a good agreement with empirical reconstructions of temperature, precipitation and vegetation for the mid-Holocene, the Last Glacial Maximum and the Last Interglacial, performing equally well as existing high-resolution snapshots of these time periods.

**Table 1:** Available reconstructions of environmental variables.

Variable	Unit	Dimensions
**Dimensional variables**		
Longitude	degrees east	720
Latitude	degrees north	300
Month	—	12
Year	before present	72
**Climatic variables**		
Monthly temperature	°C	720 × 300 × 12 × 72
Monthly precipitation	mm month^−1^	720 × 300 × 12 × 72
Monthly cloudiness	%	720 × 300 × 12 × 72
Minimum annual temperature	°C	720 × 300 × 72
Maximum annual temperature	°C	720 × 300 × 72
Monthly relative humidity	%	720 × 300 × 12 × 72
Monthly wind speed	m second^−1^	720 × 300 × 12 × 72
**Bioclimatic variables**		
BIO1: Annual mean temperature	°C	720 × 300 × 72
BIO4: Temperature seasonality	°C	720 × 300 × 72
BIO5: Minimum annual temperature	°C	720 × 300 × 72
BIO6: Maximum annual temperature	°C	720 × 300 × 72
BIO7: Temperature annual range	°C	720 × 300 × 72
BIO8: Mean temperature of the wettest quarter	°C	720 × 300 × 72
BIO9: Mean temperature of driest quarter	°C	720 × 300 × 72
BIO10: Mean temperature of warmest quarter	°C	720 × 300 × 72
BIO11: Mean temperature of coldest quarter	°C	720 × 300 × 72
BIO12: Annual precipitation	mm year^−1^	720 × 300 × 72
BIO13: Precipitation of wettest month	mm month^−1^	720 × 300 × 72
BIO14: Precipitation of driest month	mm month^−1^	720 × 300 × 72
BIO15: Precipitation seasonality	—	720 × 300 × 72
BIO16: Precipitation of wettest quarter	mm quarter^−1^	720 × 300 × 72
BIO17: Precipitation of driest quarter	mm quarter^−1^	720 × 300 × 72
BIO18: Precipitation of warmest quarter	mm quarter^−1^	720 × 300 × 72
BIO19: Precipitation of coldest quarter	mm quarter^−1^	720 × 300 × 72
**Vegetation variables**		
Net primary productivity	gC m^−2^ year^−1^	720 × 300 × 72
Leaf area index	gC m^−2^	720 × 300 × 72
Biome	categorial	720 × 300 × 72

Temperature seasonality (BIO4) and precipitation seasonality (BIO15) are given by the standard deviation of monthly temperatures and by the coefficient of variation of monthly precipitation, respectively. Temperature annual range (BIO7) is given by the difference between maximum annual temperature (BIO5) and minimum annual temperature (BIO6). Unit abbreviations: mm (millimetres), m (metres), gC (grams carbon).

## Methods

### Monthly climatic variables

Our dataset is based on simulations of monthly mean temperature (°C), precipitation (mm month^−1^), cloudiness (%), relative humidity (%) and wind speed (m s^−1^) of the HadCM3 general circulation model^6,12,13^. At a spatial grid resolution of 3.75° × 2.5°, these data cover the last 120,000 years in 72 snapshots (2,000 year time steps between 120,000 BP and 22,000 BP; 1,000 year time steps between 22,000 BP and the pre-industrial modern era), each representing climatic conditions averaged across a 30-year post-spin-up period. We denote these data by

1 $$\begin{array}{c}{T}_{{\rm{HadCM3}}}(m,t),\;{P}_{{\rm{HadCM3}}}(m,t),\;{C}_{{\rm{HadCM3}}}(m,t),\\ {H}_{{\rm{HadCM3}}}(m,t),\;{W}_{{\rm{HadCM3}}}(m,t),\end{array}$$

where $m=1,\ldots ,12$ represents a given month, and $t\in {T}_{{\rm{120}}{\rm{k}}}$ represents a given one of the 72 points in time for which simulations are available, denoted ${T}_{{\rm{120}}{\rm{k}}}$.

**Fig. 1:**
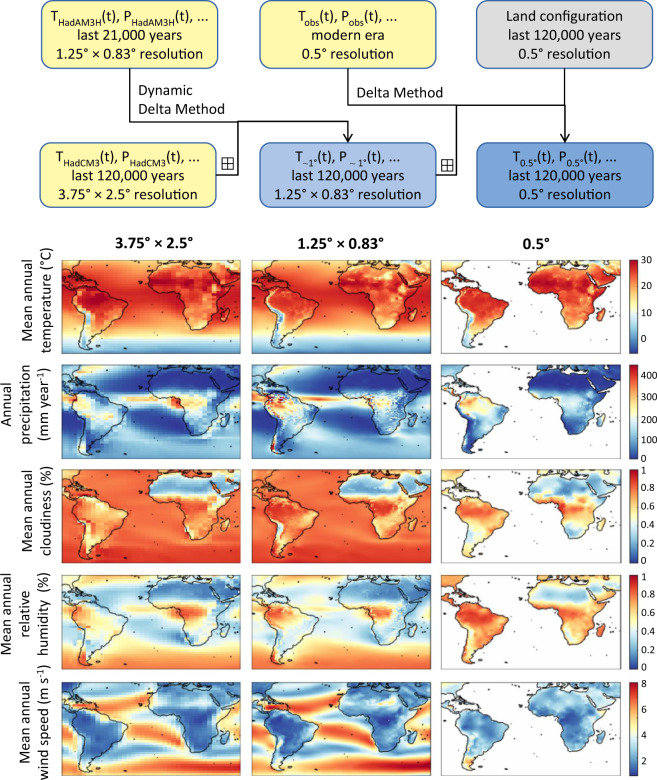
Method of reconstructing high-resolution climate. Yellow boxes represent raw simulated and observed data, the dark blue box represents the final data. Maps, showing modern-era climate, correspond to the datasets represented by the bottom three boxes.

We downscaled and bias-corrected these data in two stages (Fig. 1). Both are based on variations of the Delta Method^14^, under which a high-resolution, bias-corrected reconstruction of climate at some time *t* in the past is obtained by applying the difference between modern-era low-resolution simulated and high-resolution observed climate – the correction term – to the simulated climate at time *t*. The Delta Method has previously been used to downscale and bias-correct palaeoclimate simulations, e.g. for the widely used WorldClim database^9^. A recent evaluation of three methods commonly used for bias-correction and downscaling^15^ showed that the Delta Method reduces the difference between climate simulation data and empirical palaeoclimatic reconstructions overall more effectively than two alternative methods (statistical downscaling using Generalised Additive Models, and Quantile Mapping). We therefore used this approach for generating our dataset.

#### Downscaling to ~1° resolution

A key limitation of the Delta Method is that it assumes the modern-era correction term to be representative of past correction terms^15^. This assumption is substantially relaxed in the Dynamic Delta Method used in the first stage of our approach to downscale the data in Eq. (1) to a ~1° resolution. This involves the use of a set of high-resolution climate simulations that were run for a smaller but climatically diverse subset of ${T}_{120k}$. Simulations at this resolution are very computationally expensive, and therefore running substantially larger sets of simulations is not feasible; however, these selected data can be very effectively used to generate a suitable time-dependent correction term for each $t\in {T}_{120k}$. In this way, we are able to increase the resolution of the original climate simulations by a factor of ~9, while simultaneously allowing for temporal variability in the correction term. In the following, we detail the approach.

We used high-resolution simulations of the same variables as in Eq. (1) from the HadAM3H model^13,16,17^, available at a 1.25° × 0.83° resolution for the last 21,000 years in 9 snapshots (2,000 year time steps between 12,000 BP and 6,000 BP; 3,000 year time steps otherwise). We denote these by

$$\begin{array}{c}{T}_{{\rm{HadAM3H}}}(m,t),\;{P}_{{\rm{HadAM3H}}}(m,t),\;{C}_{{\rm{HadAM3H}}}(m,t),\\ {H}_{{\rm{HadAM3H}}}(m,t),\;{W}_{{\rm{HadAM3H}}}(m,t),\end{array}$$

respectively, where $t\in {T}_{21k}$, represents a given one of the 9 points in time for which simulations are available, denoted ${T}_{{\rm{21}}{\rm{k}}}$.

For each variable $X\in \{T,P,C,H,W\}$, we considered the differences between the medium- and the high-resolution data at times $t\in {T}_{21k}$ for which both are available,

$$\Delta {X}_{{\rm{HadAM3H}}}^{{\rm{HadCM3}}}(m,t)\,:={X}_{{\rm{HadAM3H}}}(m,t)-{X}_{{\rm{HadCM3}}}^{ \boxplus }(m,t),$$

where the $\boxplus$-notation indicates that the coarser-resolution data was interpolated to the grid of the higher-resolution data. For this, we used an Akima cubic Hermite interpolant^18^, which (unlike a bilinear interpolant) is smooth but (unlike a bicubic interpolant) avoids potential overshoots. For each $t\in {T}_{120k}$ and each $\tau \in {T}_{21k}$,

2 $${X}_{{\rm{HadCM3}}}^{ \boxplus }(m,t)+\Delta {X}_{{\rm{HadAM3H}}}^{{\rm{HadCM3}}}(m,\tau )$$

provides a 1.25° × 0.83° resolution downscaled version of the data ${X}_{{\rm{HadCM3}}}(m,t)$ in Eq. (1). The same is true, more generally, for any weighted linear combination of the Δ${X}_{{\rm{HadAM3H}}}^{{\rm{HadCM3}}}(m,\tau )$, which is the approach taken here, yielding

3 $$X{{\prime} }_{ \sim {1}^{\circ }}(m,t)\,:={X}_{{\rm{HadCM3}}}^{ \boxplus }(m,t)+\mathop{\underbrace{\sum _{\tau \in {T}_{21k}}w(t,\tau )\cdot \Delta {X}_{{\rm{HadAM3H}}}^{{\rm{HadCM3}}}(m,\tau )}}\limits_{{\rm{time-variable}}\,{\rm{correction}}\,{\rm{term}}},$$

where ${\sum }_{\tau \in {T}_{21k}}\mathop{\overbrace{w(t,\tau )}}\limits^{\ge 0}=1$ for any given $t\in {T}_{125k}$. We discuss the choice of an additive approach for all climatic variables later on. Crucially, in contrast to the classical Delta Method – which, for all $t\in {T}_{125k}$, would correspond to $w(t,{\rm{present}}\,{\rm{day}})=1$ and $w(t,\tau )=0$ otherwise (cf. Eq. (5)) –, the resolution correction term that is added to ${X}_{{\rm{HadCM3}}}^{ \boxplus }(m,t)$ in Eq. (3) need not be constant over time. Instead, the high-resolution heterogeneities that are applied to the medium-resolution HadCM3 data are chosen from the broad range of patterns simulated for ${T}_{21k}$. The strength of this approach lies in the fact that the last 21,000 years account for a substantial portion of the range of climatic conditions present during the whole Late Quaternary. Thus, by choosing the weights $w(t,\tau )$ for a given time $t\in {T}_{125k}$ appropriately, we can construct a ${T}_{21k}$-data-based correction term corresponding to a climatic state that is, in a sense yet to be specified, close to the climatic state at time *t*. Here, we used atmospheric CO_2_ concentration, a key determinant of the global climatic state^19^, as the metric according to which the $w(t,\tau )$ are chosen; i.e. we assigned a higher weight to Δ${X}_{{\rm{HadAM3H}}}^{{\rm{HadCM3}}}(m,\tau )$ the closer the CO_2_ level at time $\tau$ was to that at time *t*. Specifically, we used

$$w{\prime} (t,\tau )=\frac{1}{{({{\rm{CO}}}_{2}(t)-{{\rm{CO}}}_{2}(\tau ))}^{2}},$$

and rescaled these to $w(t,\tau )=\frac{w{\prime} (t,\tau )}{{\sum }_{\tau \in {T}_{21k}}w{\prime} (t,\tau )}$ (Supplementary Fig. 1). In the special case of $t\in {T}_{21k}$, we have $w(t,t)=1$ and $w(t,\tau )=0$ for $\tau \ne t$, for which Eq. (3) simplifies to

$$X{{\prime} }_{ \sim {1}^{\circ }}(m,t)={X}_{{\rm{HadAM3H}}}(m,t)\quad {\rm{for}}\,{\rm{all}}\,t\in {T}_{21k}.$$

Formally, the correction term in Eq. (3) corresponds to an inverse square distance interpolation of the Δ${X}_{{\rm{HadAM3H}}}^{{\rm{HadCM3}}}$ with respect to CO_2_^20^. We also note that, for our choice of $w(t,\tau )$, the correction term is a smooth function of *t*, as would be desired. In particular, this would not the case for the approach in Eq. (2) (unless $\tau$ is the same for all $t\in {T}_{125k}$).

The additive approach in Eq. (3) does not by itself ensure that the derived precipitation, relative humidity, cloudiness and wind speed are non-negative and that relative humidity and cloudiness do not exceed 100% across all points in time and space. We therefore capped values at the appropriate bounds, and obtain

4 $$\begin{array}{lll}{T}_{ \sim {1}^{\circ }}(m,t) & := & T{{\prime} }_{ \sim {1}^{\circ }}(m,t),\\ {P}_{ \sim {1}^{\circ }}(m,t) & := & {\rm{\max }}\left(0,P{{\prime} }_{ \sim {1}^{\circ }}(m,t)\right),\\ {C}_{ \sim {1}^{\circ }}(m,t) & := & {\rm{\min }}\left(100 \% ,{\rm{\max }}(0 \% ,C{{\prime} }_{ \sim {1}^{\circ }}(m,t))\right),\\ {H}_{ \sim {1}^{\circ }}(m,t) & := & {\rm{\min }}\left(100 \% ,{\rm{\max }}(0 \% ,H{{\prime} }_{ \sim {1}^{\circ }}(m,t))\right),\\ {W}_{ \sim {1}^{\circ }}(m,t) & := & {\rm{\max }}\left(0,W{{\prime} }_{ \sim {1}^{\circ }}(m,t)\right).\end{array}$$

Supplementary Fig. 2 shows that this step only affects a very small number of data points, whose values are otherwise very close to the relevant bound.

### Bias-correction and downscaling to 0.5° resolution

In the second stage of our approach, we applied the classical Delta Method to the previously downscaled simulation data. Similar to the approach in Eq. (3), this is achieved by applying a correction term, which is now given by the difference between present-era high-resolution observational climate and coarser-resolution simulated climate, to past simulated climate. This further increases the resolution and removes remaining biases in the data in Eq. (4).

Since our present-era simulation data correspond to pre-industrial conditions (280 ppm atmospheric CO_2_ concentration)^6,12,13^, it would be desirable for the observational dataset used in this step to be approximately representative of these conditions as well, so that the correction term can be computed based on the simulated and observed climate of a similar underlying scenario. There is generally a trade-off between the quality of observation-based global climate datasets of recent decades, and the extent to which they reflect anthropogenic climate change (which, by design, is not captured in our simulated data) – both of which increase towards the present. Fortunately, however, significant advances in interpolation methods^21–23^ have produced high-quality gridded datasets of global climatic conditions reaching as far back as the early 20th century^23^. Thus, here we used 0.5° resolution observational data representing 1901–1930 averages (~300 ppm atmospheric CO_2_) of terrestrial monthly temperature, precipitation and cloudiness^23^. For relative humidity and wind speed, we used a global data representing 1961–1990 average (~330 ppm atmospheric CO_2_) monthly values^24^ due to a lack of earlier datasets. We denote the data by

$${T}_{{\rm{obs}}}(m,0),\;{P}_{{\rm{obs}}}(m,0),\;{C}_{{\rm{obs}}}(m,0),\;{H}_{{\rm{obs}}}(m,0),\;{W}_{{\rm{obs}}}(m,0).$$

We extrapolated these maps to current non-land grid cells using an inverse distance weighting approach so as to be able to use the Delta Method at times of lower sea level. The resulting data were used to bias-correct and further downscale the ~1° data in Eq. (3) to a 0.5° grid resolution via

5 $$X{{\prime} }_{0.{5}^{\circ }}(m,t)\,:={X}_{ \sim {1}^{\circ }}^{ \boxplus }(m,t)+\mathop{\underbrace{{X}_{{\rm{obs}}}(m,0)-{X}_{ \sim {1}^{\circ }}^{ \boxplus }(m,0)}}\limits_{{\rm{correction}}\,{\rm{term}}},$$

where $X\in \{T,P,C,H,W\}$. In particular, the data for the present are identical to the empirically observed climate,

$$X{{\prime} }_{0.{5}^{\circ }}(m,0)={X}_{{\rm{obs}}}(m,0).$$

Finally, we again capped values at the appropriate bounds, and obtained

6a $$\begin{array}{lll}{T}_{0.{5}^{\circ }}(m,t) & := & T{{\prime} }_{0.{5}^{\circ }}(m,t),\\ {P}_{0.{5}^{\circ }}(m,t) & := & {\rm{\max }}\left(0,P{{\prime} }_{0.{5}^{\circ }}(m,t)\right),\\ {C}_{0.{5}^{\circ }}(m,t) & := & {\rm{\min }}\left(100 \% ,{\rm{\max }}(0 \% ,C{{\prime} }_{0.{5}^{\circ }}(m,t))\right),\\ {H}_{0.{5}^{\circ }}(m,t) & := & {\rm{\min }}\left(100 \% ,{\rm{\max }}(0 \% ,H{{\prime} }_{0.{5}^{\circ }}(m,t))\right),\\ {W}_{0.{5}^{\circ }}(m,t) & := & {\rm{\max }}\left(0,W{{\prime} }_{0.{5}^{\circ }}(m,t)\right).\end{array}$$

Similar as in the analogous step in the first stage of our approach (Eq. (4)), only a relatively small number of data points is affected by the capping; their values are reasonably close to the relevant bounds, and their frequency decreases sharply with increasing distance to the bounds (Supplementary Fig. 2).

In principle, capping values, where necessary, can be circumvented by suitably transforming the relevant variable first, then applying the additive Delta Method, and back-transforming the result. In the case of precipitation, for example, a log-transformation is sometimes used, which is mathematically equivalent to a multiplicative Delta Method, in which low-resolution past simulated data is multiplied by the relative difference between high- and low-resolution modern-era data^14^; thus, instead of Eq. (5), we would have ${P}_{0.{5}^{\circ }}(m,t)\,:={P}_{ \sim {1}^{\circ }}^{ \boxplus }(m,t)\cdot \frac{{P}_{{\rm{obs}}}(m,0)}{{P}_{ \sim {1}^{\circ }}^{ \boxplus }(m,0)}$. However, whilst this approach ensures non-negative values, it has three important drawbacks. First, if present-era observed precipitation in a certain month and grid cell is zero, i.e. ${P}_{{\rm{obs}}}(m,0)=0$, then ${P}_{0.{5}^{\circ }}(m,t)=0$ at all points in time, *t*, irrespectively of the simulated climate change signal. Specifically, this makes it impossible for current extreme desert areas to be wetter at any point in the past. Second, if present-era simulated precipitation in a grid cell is very low (or indeed identical to zero), i.e. ${P}_{ \sim {1}^{\circ }}^{ \boxplus }(m,0)\approx 0$, then ${P}_{0.{5}^{\circ }}(m,t)$ can increase beyond all bounds. Very arid locations are particularly prone to this effect, which can generate highly improbable precipitation patterns for the past. In our scenario of generating global maps for a total of 864 individual months, this lack of robustness of the multiplicative Delta Method would be difficult to handle. Third, the multiplicative Delta Method is not self-consistent: applying it to the sum of simulated monthly precipitation does not produce the same result as applying it to simulated monthly precipitation first and then taking the sum of these values. The natural equivalent of the log-transformation for precipitation is the logit-transformation for cloudiness and relative humidity, however, this approach suffers from the same drawbacks.

### Minimum and maximum annual temperature

Diurnal temperature data are not included in the available HadCM3 and HadAM3H simulation outputs. We therefore used the following approach to estimate minimum and maximum annual temperatures. Based on the monthly HadCM3 and HadAM3H temperature data, we created maps of the mean temperature of the coldest and the warmest month. In the same way as described above, we used these data to reconstruct the mean temperature of the coldest and warmest month at a 1.25° × 0.83° resolution by means of the Dynamic Delta Method, yielding

$${T}_{ \sim {1}^{\circ }}^{{\rm{coldest}}\,{\rm{month}}}(t)\,{\rm{and}}\,{T}_{ \sim {1}^{\circ }}^{{\rm{warmest}}\,{\rm{month}}}(t),$$

for $t\in {T}_{120k}$. We then used observation-based 0.5° resolution global datasets of modern-era (1901–1930 average) minimum and maximum annual temperature^23^, denoted

$${T}_{{\rm{obs}}}^{{\rm{\min }}}(0)\,{\rm{and}}\,{T}_{{\rm{obs}}}^{{\rm{\max }}}(0),$$

to estimate past minimum and maximum annual temperature as

6b $$\begin{array}{lll}{T}_{0.{5}^{\circ }}^{{\rm{\min }}}(t) & := & {T}_{ \sim {1}^{\circ }}^{{\rm{coldest}}\,{\rm{month}}, \boxplus }(t)+{T}_{{\rm{obs}}}^{{\rm{\min }}}(0)-{T}_{ \sim {1}^{\circ }}^{{\rm{coldest}}\,{\rm{month}}, \boxplus }(0),\\ {T}_{0.{5}^{\circ }}^{{\rm{\max }}}(t) & := & {T}_{ \sim {1}^{\circ }}^{{\rm{warmest}}\,{\rm{month}}, \boxplus }(t)+{T}_{{\rm{obs}}}^{{\rm{\max }}}(0)-{T}_{ \sim {1}^{\circ }}^{{\rm{warmest}}\,{\rm{month}}, \boxplus }(0),\end{array}$$

respectively. This approach assumes that the difference between past and present mean temperature of the coldest (warmest) month is similar to the difference between the past and present temperature of the coldest (warmest) day. Instrumental data of the recent past suggest that this assumption is well justified across space (Supplementary Fig. 3).

### Land configuration

We used a reconstruction of mean global sea level^25^ and a global elevation and bathymetry map^26^, interpolated to a 0.5° resolution grid, to create land configuration maps for the last 120,000 years. Maps of terrestrial climate through time were obtained by cropping the global data in Eq. (6a and b) to the appropriate land masks. Values in non-land grid cells were set to missing values, except in the case of below-sea-level inland grid cells, such as the Aral, Caspian and Dead sea.

### Bioclimatic data, net primary productivity, leaf area index, biome

Based on our reconstructions of minimum and maximum annual temperature, and monthly temperature and precipitation, we derived 17 bioclimatic variables^27^ listed in Table 1. In addition, we used the Biome4 global vegetation model^28^ to compute net primary productivity, leaf area index and biome type at a 0.5° resolution for all $t\in {T}_{120k}$, using reconstructed minimum annual temperature, and monthly temperature, precipitation and cloudiness. Similar to a previous approach^21^, we converted cloudiness to the percent of possible sunshine (required by Biome4) by using a standard conversion table and applying an additional latitude- and month-specific correction. Since Biome4 estimates ice biomes only based on climatic conditions and not ice sheet data, it can underestimate the spatial extent of ice. We therefore changed simulated non-ice biomes to ice, and set net primary production and leaf area index to 0, in grid cells covered by ice sheets according to the ICE-6g dataset^29^ at the relevant points in time. Whilst our data represent potential natural biomes, and as such do not account for local anthropogenic land use, maps of actual land cover can readily be generated by superimposing our data with available reconstructions of global land use during the Holocene^30^.

## Data Records

Our dataset, containing the variables listed in Table 1, is available as a single NetCDF file on the Figshare data repository^31^. All maps are provided at 2,000 year time steps between 120,000 BP and 22,000 BP, and 1,000 year time steps between 22,000 BP and the (pre-industrial) modern era. We used a 0.5° equirectangular grid, with longitudes ranging between 179.75°E and 179.75°W, and latitudes ranging between 59.75°S and 89.75°N.

## Technical Validation

**Fig. 2:**
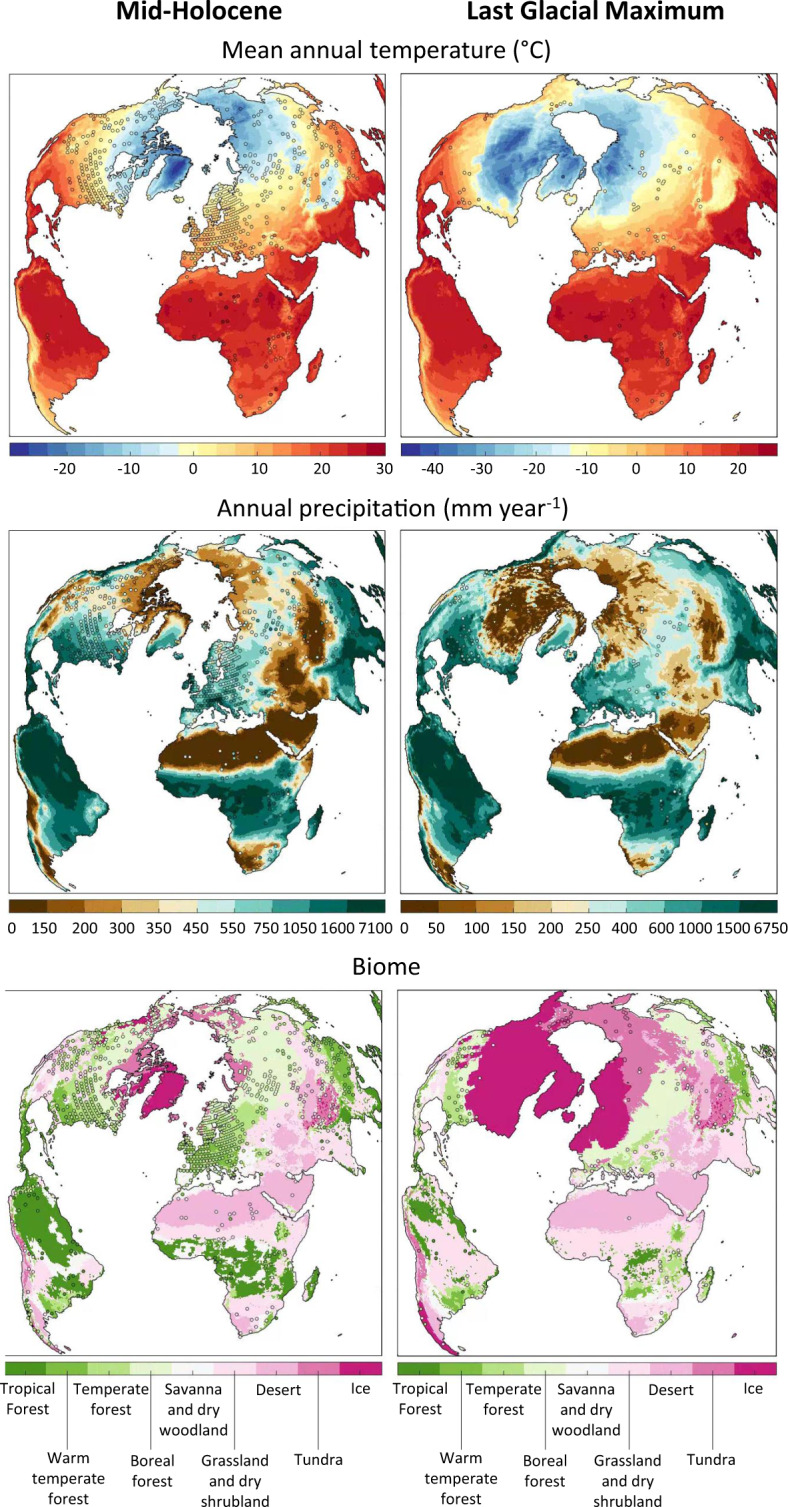
Comparison between modelled mid-Holocene and Last Glacial Maximum temperature, precipitation and vegetation (maps), and pollen-based empirical reconstructions (markers; uncertainties not shown)^32,34^. For visualisation purposes, empirical biomes were aggregated to a 2° grid, and the set of 27 simulated biomes was grouped into 9 megabiomes.

**Fig. 3:**
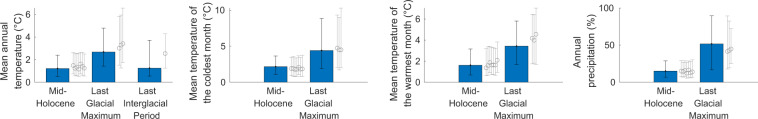
Quantitative comparison between our data and empirical reconstructions of available climatic variables^32,33^, and data from other climate models. Blue bars and black error bars represent the median and the upper and lower quartiles of the set of absolute differences between our data and the available empirical reconstructions (cf.^15^ for details). Supplementary Fig. 4 shows all individual data points that these summary statistics are based on. Grey error bars show the equivalent measures for palaeoclimate data available on WorldClim v1.4^9^, i.e. from the IPSL-CM5A-LR, MRI-CGCM3, BCC-CSM1-1, CNRM-CM5 and CCSM4 models (Mid-Holocene), the MPI-ESM-P and MIROC-ESM models (Mid-Holocene and Last Glacial Maximum) and the CCSM4 model (Last Glacial Maximum and Last Interglacial Period).

Proxy data-based reconstructions of past climatologies allow us to evaluate our dataset by means of empirical records, and compare its performance against that of existing model-based snapshots of specific time periods. Here, we used empirical reconstructions of mean annual temperature, temperature of the coldest and warmest month, and annual precipitation for the Mid-Holocene and the Last Glacial Maximum^32^, and reconstructions of mean annual temperature for the Last Interglacial Period^33^. Overall, our data are in good agreement with the available empirical reconstructions (Fig. 2, Supplementary Fig. 4). For each variable and time period, residual biases across the value spectrum are approximately normally distributed around zero, with the possible exception of precipitation, where, at the lower end of the value spectrum, a few empirical reconstructions suggest slightly higher values than our dataset (Supplementary Fig. 4). By construction of the Delta Method, our modern-era data is identical to the observed climate. Simulated vegetation, a product of temperature, precipitation and cloud cover data, also corresponds well to empirical biome reconstructions available for the Mid-Holocene and Last Glacial Maximum^34^ (Fig. 2). The performance of our data under the available empirical palaeoclimatic reconstructions is well within the range of that of downscaled and bias-corrected outputs from other climate models available for specific points in the past (Fig. 3).

## Usage Notes

The dataset comes with fully commented R, Python and Matlab scripts that demonstrate how annual and monthly variables can be read from the NetCDF file, and how climatic data for specific points in time and space can be extracted and analysed.

## Supplementary information

Supplementary Figures

## Data Availability

Code used to generate our dataset is available on the Open Science Framework^35^.
