# Addendum: High-resolution terrestrial climate, bioclimate and vegetation for the last 120,000 years

**DOI:** 10.1038/s41597-021-01051-1

**Published:** 2021-09-29

**Authors:** Robert M. Beyer, Mario Krapp, Andrea Manica

**Affiliations:** Department of Zoology, University of Cambridge, Downing Street, Cambridge, CB2 3EJ United Kingdom

**Keywords:** Palaeoecology, Palaeoclimate

Addendum to: *Scientific Data* 10.1038/s41597-020-0552-1, published online 14 July 2020

Our original dataset^1^ provides estimates of annual net primary productivity (NPP). Whilst this variable can be a useful proxy for the resource availability in ecosystems across a full annual cycle, some applications at palaeo time scales require reconstructions of vegetation dynamics at a higher temporal resolution, for example when examining seasonal species distribution and migration^2^. In the updated version of our dataset, we therefore additionally provide late Quaternary global estimates of monthly NPP.

The default output of Biome4^3^, the vegetation model used to generate our original dataset, includes only annual NPP; however, monthly NPP values are computed internally. We modified the Biome4 code to include these values in the output variables^4^. The derived monthly NPP maps are available with the original dataset on the Figshare repository^5^. They are provided in the same format as the monthly climate data (i.e., covering 720 longitudinal degrees, 300 latitudinal degrees, 12 months, and 72 years) and can be read and analysed using the R, Python, and Matlab scripts included in the original dataset.
