# Supplementary material for: High-resolution terrestrial climate, bioclimate and vegetation for the last 120,000 years
Source: Sci Data. 2020 Jul 14;7:236. doi: 10.1038/s41597-020-0552-1 (PMC7360617; doi:10.1038/s41597-020-0552-1)
Supplement: Supplementary file 1 — Supplementary Figures [file 41597_2020_552_MOESM1_ESM.pdf]

Supplementary Files

Table of Contents

Supplementary Figure 1 – Page 1
Supplementary Figure 2 – Page 2
Supplementary Figure 3 – Page 3
Supplementary Figure 4 – Page 4

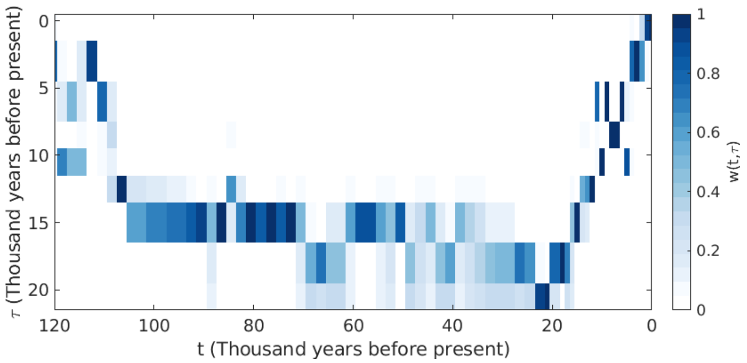

Supplementary Figure 1: Visualisation of the weights $w(t, \tau)$ used in Eq. (3).

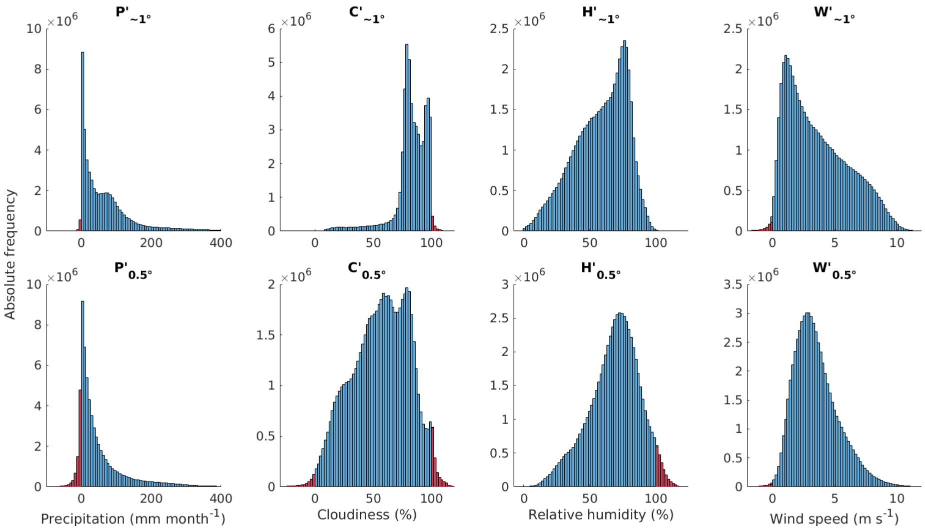

Supplementary Figure 2: Distribution of values (combined from all years, months and grid cells) of $X'_{\sim 1^\circ}$ in Eq. (3) (top row), and $X'_{0.5^\circ}$ in Eq. (5) (bottom row), i.e. prior to the capping in Eqs. (4) and (6a), respectively, where values corresponding to red bins are set to the appropriate minimum or maximum of the allowable range. (Note that the data shown in the bottom row correspond to a finer spatial grid and do not include non-land grid cells.)

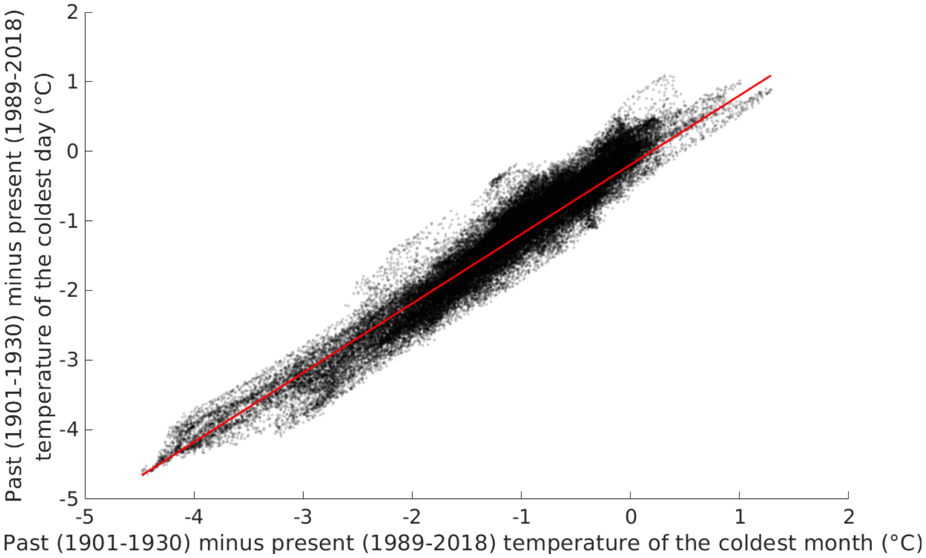

Supplementary Figure 3: Comparison between (x-axis) the difference between past and present average temperature of the coldest month, and (y-axis) the difference between past and present temperature of the coldest day of the year. Past and present data, respectively, represent averages of the first and the last 30-year period (1901–1930 and 1989–2018) of a global observation-based dataset [22]. Markers represent the set of all land points on a 0.5° spatial grid. The red line shows the 1:1 relationship. The mean absolute difference between x- and y-coordinates is 0.2°C.

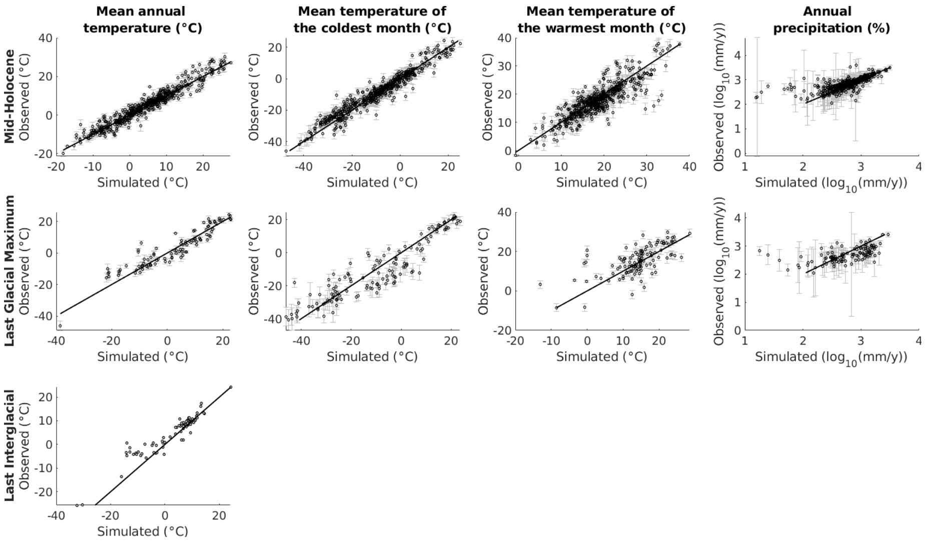

Supplementary Figure 4: Comparison between our data and available empirically reconstructed climate variables [31, 32], showing all data points and empirical standard errors (where available). Black lines represent 1:1 relationships.
